# Rhomboid family member 2 regulates cytoskeletal stress-associated Keratin 16

**DOI:** 10.1038/ncomms14174

**Published:** 2017-01-27

**Authors:** Thiviyani Maruthappu, Anissa Chikh, Benjamin Fell, Paul J. Delaney, Matthew A. Brooke, Clemence Levet, Angela Moncada-Pazos, Akemi Ishida-Yamamoto, Diana Blaydon, Ahmad Waseem, Irene M. Leigh, Matthew Freeman, David P. Kelsell

**Affiliations:** 1 Centre for Cell Biology and Cutaneous Research, Blizard Institute, Barts and the London School of Medicine and Dentistry, Queen Mary University of London, 4 Newark Street, Whitechapel London E1 2AT, UK; 2 Sir William Dunn School of Pathology, South Parks Road, Oxford OX1 3RE, UK; 3 Department of Dermatology, Asahikawa Medical University, Asahikawa 078-8510, Japan; 4 Centre for Clinical and Diagnostic Oral Sciences, Institute of Dentistry, Barts and the London School of Medicine and Dentistry, Queen Mary University of London, London E1 2AT, UK; 5 Centre for Centre Molecular Medicine, Clinical Research Centre, Ninewells Hospital and Medical School, Dundee DD1 9SY, UK

## Abstract

Keratin 16 (K16) is a cytoskeletal scaffolding protein highly expressed at pressure-bearing sites of the mammalian footpad. It can be induced in hyperproliferative states such as wound healing, inflammation and cancer. Here we show that the inactive rhomboid protease RHBDF2 (iRHOM2) regulates thickening of the footpad epidermis through its interaction with K16. K16 expression is absent in the thinned footpads of *irhom2*^−/−^ mice compared with *irhom2*^*+/+*^mice, due to reduced keratinocyte proliferation. Gain-of-function mutations in iRHOM2 underlie Tylosis with oesophageal cancer (TOC), characterized by palmoplantar thickening, upregulate K16 with robust downregulation of its type II keratin binding partner, K6. By orchestrating the remodelling and turnover of K16, and uncoupling it from K6, iRHOM2 regulates the epithelial response to physical stress. These findings contribute to our understanding of the molecular mechanisms underlying hyperproliferation of the palmoplantar epidermis in both physiological and disease states, and how this ‘stress' keratin is regulated.

## Abstract (web summary)

Keratin 16 is an epithelial protein highly expressed at pressure bearing sites and during wound healing and cancer. Here the authors show that K16 interacts with the inactive protease Rhbdf2, associated with Tylosis with oesophageal cancer, and that this interaction drives increased keratinocyte proliferation.

The ridged skin of the palms and soles (palmoplantar sites) in mammals is uniquely adapted to withstand remarkable physical stress^1^,^2^,^3^,^4^. The actual physical resilience of the palmoplantar epidermis is thought to be predominantly due to an abundance of cytoskeletal scaffolding proteins known as keratin intermediate filaments^5^. Keratins constitute up to 85% of the protein content of differentiated epithelial cells and provide both flexibility and strength by forming a framework within the cytoplasm^6^. Over 50 Keratin isotypes are expressed in stratified and pseudostratified epithelia^7^; however, their precise expression pattern is both site and situation specific^8^,^9^. Keratins are also expressed in a pairwise manner, with a type I keratin (acidic, keratins 9–20) paired with its specific type II partner (basic, keratins 1–8), for example, K6/K16 and K1/K10 (refs 8, 10). Specific pairs initiate heterodimerization through their coiled-coil domains, forming 10 nm filaments, which self-assemble to form the keratin filament network^11^,^12^.

Unlike inter-follicular skin, the ridged skin of the palms and soles is characterized by the constitutive expression of Keratins 6, 16, 17 and uniquely, Keratin 9 (K9)^1^,^5^. In addition to its high expression in the palmoplantar epidermis, K16 can be induced in hyperproliferative states such as during wound healing^2^,^3^ and inflammatory skin diseases^13^. On regeneration, epithelial cells at the wound edge deviate from their normal expression pattern, downregulating K10 and inducing K16 expression within 6 h of injury^2^,^3^. K16 is also a biomarker of several cancers particularly squamous cell carcinomas, including those originating from cervical, oesophageal and nasopharyngeal epithelia^9^,^14^,^15^. The tightly restricted expression pattern of K16 supports the notion that this particular keratin bears unique properties; however, how the dynamic regulation of K16 occurs in response to physiological and cellular stress remains poorly understood.

Several genetic skin disorders show a predilection for palmoplantar sites^16^. For example, germline dominant or recessive mutations in K16 in both humans^17^,^18^ and mice^19^ lead to hyperproliferative thickening of the palms and soles termed palmoplantar keratoderma (PPK). In humans, PPKs arising from dominantly inherited mutations in K16 are called pachyonychia congenita. This monogenic disorder is associated with painful thickening of the palmoplantar epidermis on weight bearing^17^. Other PPKs can be caused by inherited defects in non-keratin genes^16^. One example is Tylosis with oesophageal cancer (TOC, OMIM:148500)^20^. TOC is inherited as an autosomal dominant trait and affected individuals first present with PPK in childhood^21^. Moreover, they carry a >95% lifetime risk of developing oesophageal squamous cell carcinoma by 65 years of age^21^. Gain-of-function mutations in the gene encoding the inactive rhomboid protease RHBDF2 (iRHOM2) are the underlying cause of this syndrome^4^. In mammalian myeloid and epithelial cells, iRHOM2 promotes the maturation and trafficking of ADAM17 (a disintegrin and metalloproteinase-17, or tumour necrosis factor-α (TNFα)-converting enzyme) in the endoplasmic reticulum and Golgi^22^. Activated ADAM17 then transits to the plasma membrane where it is able to cleave its substrates including TNFα, interleukin-6R and epidermal growth factor receptor family ligands^23^,^24^. In TOC-derived keratinocytes, *iRHOM2* mutations lead to high levels of constitutive ADAM17 activity with elevated ectodomain shedding of many of its substrates^23^. TOC keratinocytes also show increased rates of proliferation and rapid migration in monolayer scratch-wound assays when compared with control keratinocytes^4^. These features suggest that TOC keratinocytes exhibit features resembling a state of constitutive wound healing. Furthermore, Phorbol 12-myristate 13-acetate (PMA)-stimulated peripheral blood mononuclear cells (PBMCs) from TOC patients also have increased TNFα release compared with control PBMCs^23^. In contrast, PBMCs/macrophages from *irhom2*^−/−^ mice have impaired ADAM17 maturation and, consequently, TNFα shedding from these cells is almost entirely abolished in response to bacterial stressors^22^,^25^.

In this study, we identify iRHOM2 as a novel regulator of K16 in humans and mice, with important implications for PPKs, wound healing, inflammatory skin disease and cancers. We demonstrate for the first time that iRHOM2 binds to K16, and that this interaction is increased in TOC keratinocytes, where it is associated with robust downregulation of K16's binding partner K6. In humans and mice, iRHOM2 loss results in dampened proliferation and reduced K16 expression, and in mice this leads to thinning of epidermis in the footpad. These findings highlight a novel and fundamental role for iRHOM2 in regulating the epithelial response to mechanical stress.

## Results

### *iRHOM2* ^*−/−*^ mice paws reveal a thinner epidermis

**Figure 1:**
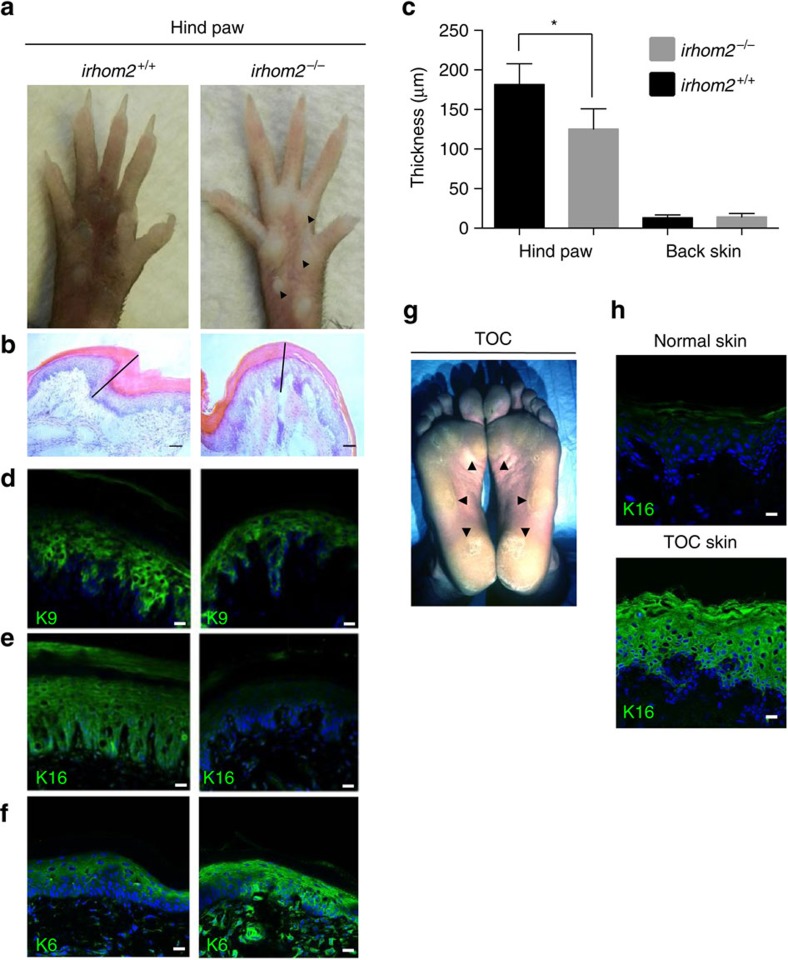
*irhom2*^−/−^ mice exhibit reduction in epidermal thickness. (**a**) The hind paws of 16-week-old *irhom2*^−/−^ and *irhom2*^*+/+*^ littermates (*n*=4 per genotype) were photographed, revealing pallor and diminution of the major stress-bearing paw footpads calluses (black arrowhead) in adult *irhom2*^−/−^ mice. (**b**) Haematoxylin and eosin-stained cross-sections of hind-paw footpads showed a reduction in thickness of the epidermis (black line) in *irhom2*^−/−^ mice. Scale bars, 50 μm. (**c**) Graph depicts the mean thickness of the epidermis of hind paw and back skin from *irhom2*^*+/+*^ and *irhom2*^−/−^ mice calculated from ten individual measurements in five separate cross-sections from each genotype (*n*=3); error bars denote s.d., *P<0.05 (Student's *t*-test). (**d**) Immunohistochemistry showing similar K9 expression in paw skin sections. Scale bars, 20 μm. (**e**) Immunohistochemistry demonstrating reduction of K16 expression in paw skin sections. Scale bars, 20 μm. (**f**) Immunohistochemistry showing upregulation of K6 expression in the hind paw epidermis of *irhom2*^−/−^ mice compared with *irhom2*^*+/+*^. (**g**) TOC patient feet showing symmetrically thickened skin at pressure-bearing sites (black arrowheads) on the plantar epidermis. (**h**) TOC skin demonstrates increased K16 expression throughout the epidermis in comparison with control inter-follicular skin. Scale bars, 20 μm.

To elucidate the role of iRHOM2 in the skin, we examined *irhom2*^−/−^ mice^22^,^25^. Although these mice were reported to be physically similar to *irhom2*^*+/+*^ mice^20^,^23^, re-assessment revealed a striking difference in the paws of adult *irhom2*^−/−^ mice. Examination of their forepaws and hind paws revealed diminution of the stress-bearing footpads and also showed pallor and translucency, lacking the normal thickening and hyperpigmentation of the footpads seen in *irhom2*^*+/+*^ littermates (Fig. 1a and Supplementary Fig. 1a). Microscopic examination of haematoxylin and eosin-stained skin sections of the fore and hind paws of *irhom2*^−/−^ mice showed thinning of the epidermis (Fig. 1b and Supplementary Fig. 1a). Quantification of epidermal thickness demonstrated that *irhom2*^−/−^ mice footpads were significantly thinner than *irhom2*^*+/+*^ (Fig. 1c). However, there was no significant difference in the thickness of their back skin. As keratins comprise a major component of the footpad epidermis and are known to be altered in palmoplanter keratodermas^16^, we performed immunohistochemical staining for the palmoplantar-expressed keratins; K6, K16 and K9. Surprisingly, this revealed diminished K16 expression in *irhom2*^−/−^ throughout the footpad epidermis compared with *irhom2*^*+/+*^ controls (Fig. 1d and Supplementary Fig. 1d), whereas the expression of K9 was comparable (Fig. 1e and Supplementary Fig. 1e). K6, the predominant type II binding partner of K16 however appeared to be upregulated compared with *irhom2*^*+/+*^ (Fig. 1f and Supplementary Fig. 1f). The *irhom2*^−/−^ mouse footpad phenotype directly contrasts what is observed in TOC plantar skin, which exhibits marked thickening and callous formation at sites of pressure (Fig. 1g). Consistent with the opposing skin phenotypes, the expression pattern of keratins was also reversed in TOC skin compared with *irhom2*^−/−^ mice, with increased K16 expression in lesional interfollicular (non-palm) epidermis (Fig. 1h). These findings implicate iRHOM2 in determining footpad thickness and K16 expression in humans and mice.

### K16 is a novel interacting binding partner of iRHOM2

**Figure 2:**
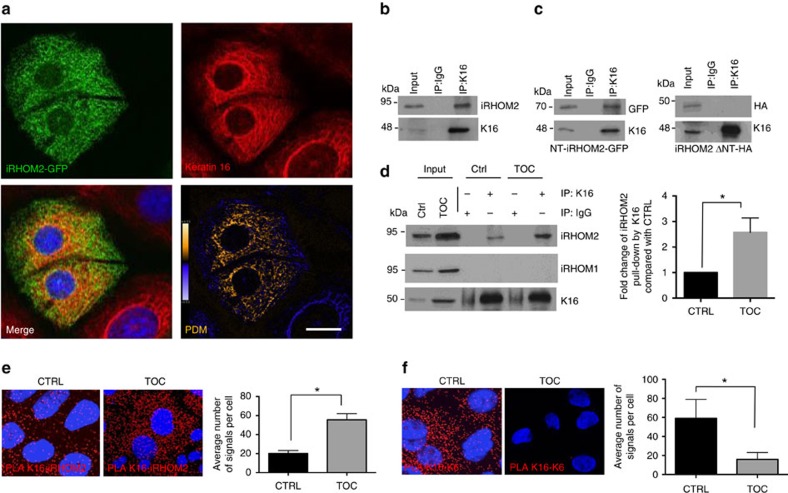
The N terminus of iRHOM2 interacts with K16. (**a**) Confocal microscopy analysis of GFP-tagged iRHOM2 overexpressed in CTRL keratinocytes showing co-localization with endogenous K16. Pseudocoloured ‘product of the differences from the mean' (PDM) images, in which each pixel is equal to the PDM value at that location, are shown with a PDM scale. Scale bar, 20 μm. (**b**) Control (CTRL) keratinocyte lysates were immunoprecipitated with anti-K16 antibody and immunoblotted with anti-iRHOM2 antibody. (**c**) Left panel: cell extracts from CTRL keratinocytes transfected with a vector expressing NT-iRHOM2-GFP were immunoprecipitated with anti-K16 antibody and analysed by western blotting with anti-GFP and anti-K16 antibodies. Right panel: cell extracts from CTRL keratinocytes transfected with a vector expressing HA-tagged iRHOM2 lacking its N terminus (iRHOM2-ΔNT-HA) were immunoprecipitated with anti-K16 antibody and analysed by western blotting with anti-HA and anti-K16 antibodies. (**d**) CTRL and TOC keratinocyte lysates were immunoprecipitated with anti-K16 antibody and analysed by western blotting with anti-iRHOM1 and anti-iRHOM2 antibody. Graph depicting fold change of iRHOM2 pull-down by K16 in TOC cells compared with CTRL (*n*=3); error bars denote s.d., *P<0.05 (Student's *t*-test). (**e**) PLA between iRHOM2 and K16 analysed by confocal microscopy in CTRL and TOC keratinocytes. Graph represents quantification of red fluorescent PLA signals relative to 4,6-diamidino-2-phenylindole (DAPI). Mean of 3 experiments, each experiment comprising 12 separate images. Error bars denote s.d. *P<0.05 (Student's *t*-test). Scale bar, 20 μm. (**f**) PLA between K6 and K16 analysed by confocal microscopy. The graph shows quantification of red fluorescent PLA signals relative to DAPI. Mean of 3 experiments, each experiment comprising 12 separate images. Error bars denote s.d.*P<0.05 (Student's *t*-test). Scale bar, 20 μm.

To decipher how iRHOM2 might regulate the opposing cutaneous phenotypes in mice and men, and in particular the differences in K16 expression, a yeast two-hybrid screen was performed using iRHOM2 as bait (aa1–403) in a three-dimensional (3D) reconstituted skin library. The cytoplasmic amino-terminus domain of iRHOM2 was used as bait in the screen, because all described TOC-causing mutations (p.Ile186Thr, p.Asp188Ans and p.Pro189Leu) occur within four residues of this highly conserved region unique to the iRHOMs^4^,^26^. This screen uncovered K16 as a highly plausible interacting protein (Supplementary Fig. 1g). Immunocytochemistry revealed partial colocalisation between green fluorescent protein (GFP)-tagged iRHOM2 and endogenous K16 in the human immortalized HaCaT keratinocytes cell line (Fig. 2a). To confirm the proposed interaction we performed co-immunoprecipitation, which showed that endogenous K16 was able to efficiently immunoprecipitate iRHOM2 (Fig. 2b) in HaCaT cells. To further validate the results of the yeast two-hybrid screen, the GFP-tagged N-terminus domain of iRHOM2 or iRHOM2 lacking its N-terminus were overexpressed in HaCaTs. K16 was able to immunoprecipitate the N terminus of iRHOM2 but not when this domain was absent (Fig. 2c). The GFP empty vector control was negative (Supplementary Fig. 2a). These data confirm the results of the yeast two-hybrid screen and indicate that iRHOM2 binds K16, and that this occurs through its N-terminus domain.

As all TOC-causing mutations occur within the N-terminus, we considered whether this could affect its binding with K16 and contribute to the high levels of K16 observed in the TOC epidermis. Immortalized TOC keratinocytes originally derived from TOC individuals with the dominantly inherited iRHOM2 mutation p.Il2186Thr were used in subsequent experiments^4^. Endogenous co-immunoprecipitation using K16 to pull-down iRHOM2 demonstrated significantly increased interaction in TOC keratinocytes (TOC) compared with similarly immortalized control keratinocytes (CTRL; Fig. 2d). It has previously been reported that both iRHOM2 and its rhomboid family member iRHOM1 can bind ADAM17 (ref. 27); however, K16 did not bind iRHOM1 in pull-down assays using TOC and CTRL cells (Fig. 2d and Supplementary Fig. 2b), suggesting that, unlike ADAM17, the interaction with K16 is iRHOM2 specific. We also tested whether iRHOM2 could pull down another abundant cytoskeletal protein, Vinculin and did not observe an interaction (Supplementary Fig. 2c).

To further quantify the differences in iRHOM2-K16 binding between CTRL and TOC cells, proximity ligation assay (PLA) was performed. This technique yields a signal when two proteins of interest are within 40 nm of each other and confirmed significantly increased iRHOM2-K16 signal frequency in TOC keratinocytes (Fig. 2e). Thus, TOC-associated *iRHOM2* mutations appear to increase its binding with K16. As we observed that K6 expression was increased in the epidermis of *irhom2*^−/−^ mice, despite a reduction in K16, PLA was also performed between K6 and K16. This showed a significant reduction in the interaction between the two known binding partners in TOC cells (Fig. 2f). Taken together, these data indicate that there is a shift in the balance between the binding partners from K16–K6 to K16–iRHOM2 in the presence of increased iRHOM2 activity in TOC.

### K16 and K6 are differentially regulated in TOC

**Figure 3:**
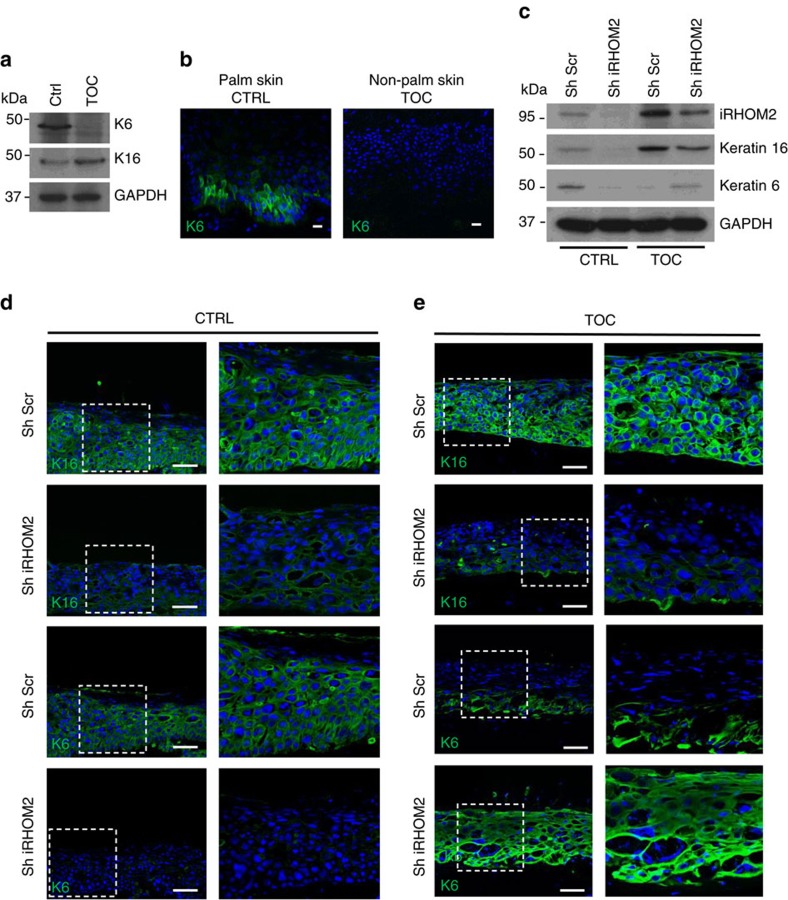
K16 and K6 are differentially regulated in TOC. (**a**) Western blot analysis of endogenous expression of K16 and K6 in CTRL and TOC keratinocytes. GAPDH was used as a loading control. (**b**) Confocal analysis performed on palm skin (positive control) and non-palmar TOC skin shows diminished K6 expression in the TOC epidermis. Scale bars, 20 μm. (**c**) Western blot analysis showing that IRHOM2 depletion by shRNA reduces the expression of K16 in CTRL and TOC keratinocytes, while it restores expression of K6 in TOC keratinocytes. GAPDH was used as a loading control. (**d**) Confocal analysis showing the reduction of K16 and K6 expression in Sh-iRHOM2 CTRL 3D human skin equivalents. Scale bars, 20 μm. (**e**) Confocal analysis showing the reduction of K16 expression but upregulation of K6 in Sh-iRHOM2 TOC 3D human skin equivalents. Scale bars, 20 μm.

K16 is a type I keratin that forms heterodimers with its major type II binding partner K6 (ref. 8). Western blotting confirmed a reduction of K6 in TOC keratinocytes compared with CTRL (Fig. 3a). In addition, immunohistochemistry of TOC skin showed almost absent K6, despite the presence of abundant K16 (Fig. 3b). Confocal analysis of TOC cells showed poorly formed K6 filaments that were reduced to cytoplasmic aggregates compared with the branch-like filament network observed in CTRL keratinocytes (Supplementary Fig. 2d). Furthermore, short hairpin RNA (shRNA) knockdown of iRHOM2 in TOC cells resulted in restoration of K6 levels comparable to CTRL (Fig. 3c). Re-expression of K6 was also shown in 3D human skin equivalents derived from sh-iRHOM2 TOC cells, in contrast to reduced K6 expression in CTRL 3D cultures, whereas K16 was reduced in both sh-iRHOM2 cell lines (Fig. 3d,e). Intriguingly, quantitative PCR showed that K6 messenger RNA (mRNA) levels were significantly reduced in TOC cells, where K16 mRNA levels were increased, suggesting that the regulation of these two keratins also occurs at transcriptional level (Supplementary Fig. 2e). These data imply that the gain-of-function mutant form of iRHOM2 in TOC is associated with increased interaction with K16 and reduced levels of K6 at both mRNA and protein level, suggesting a reciprocal regulation of K6 and K16. To explore a potential mechanism for this, we re-evaluated the yeast two-hybrid screen. The two prey clone K16 fragments found to interact with the iRHOM2 N-terminus bait share a 143 amino acid overlapping region, which corresponds to Coil 1B of K16 (Supplementary Fig. 3a). Keratins are composed of head and tail domains, as well as central helical coiled-coiled domains (Coil 1A, 1B 2A and 2B), each with specific functions^28^. The Coil 1B domain plays a role in heterodimerisation between type I and II keratins^29^. Increased binding of iRHOM2 to the coil 1B domain of K16 in TOC could therefore physically impair dimerization with its binding partner K6 and potentially contribute to its downregulation. Our data imply that differential modulation of K16 and K6 expression is likely to be iRHOM2 dependent.

### iRHOM2 regulates the reorganization and dynamicity of K16

**Figure 4:**
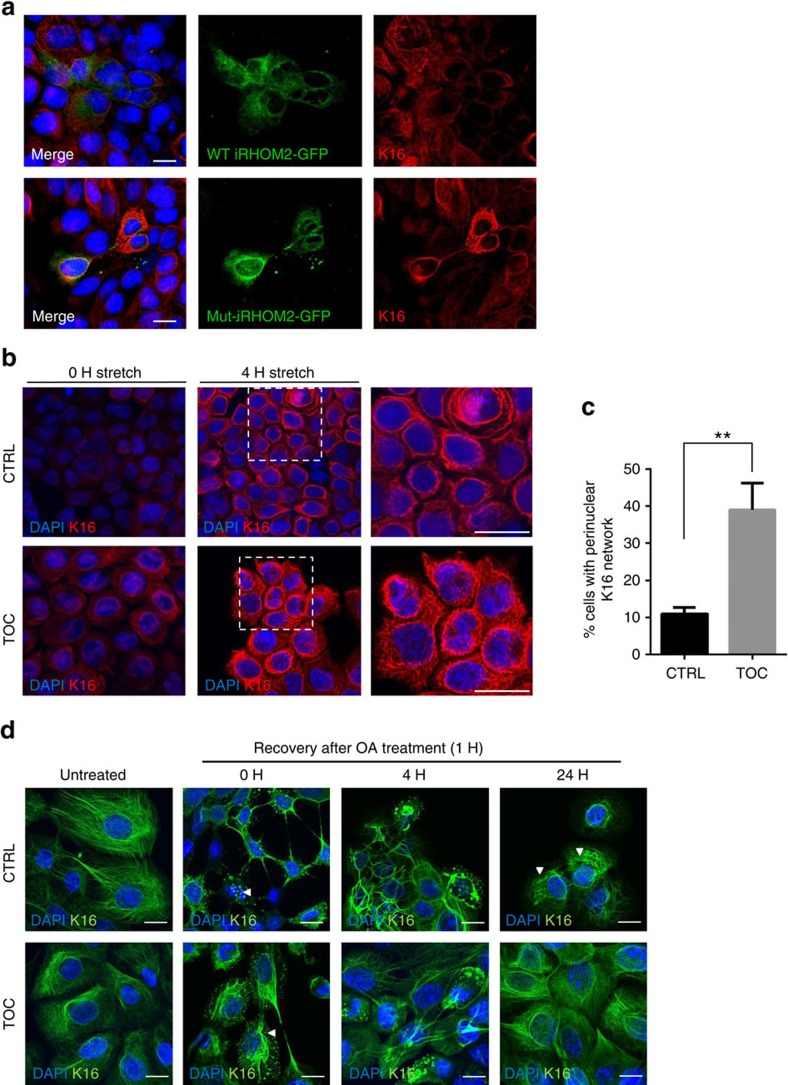
iRHOM2 regulates the reorganization and dynamicity of K16 filaments. (**a**) CTRL keratinocytes were transfected with either WT-iRHOM2-GFP or TOC Mutant-iRHOM2-GFP and immunostained for endogenous K16 and analysed by confocal microscopy. Scale bar, 20 μm. (**b**) CTRL and TOC keratinocytes were subjected to cyclical mechanical stretch at a frequency of 5 Hz and amplitude 10–13% using Flexcell FX-4000 Tension system for 0 and 4 h, respectively. Stretched cells were immunostained for K16 and analysed by confocal microscopy. White arrowheads indicate perinuclear K16 filaments reorganization. Scale bar, 20 μm. (**c**) Quantification of the perinuclear localization of K16 filaments in CTRL and TOC cells. Data represent the mean of 3 independent experiments, each experiment comprising 30 images per cell line. Error bars denote s.d. **P<0.01 (Student's *t*-test). (**d**) Confocal analysis of CTRL and TOC keratinocytes treated with 1 μM OA for 1 h, then fixed 4 and 24 h following treatment and immunostained for K16. White arrowheads represent aggregated K16 filaments. Scale bar, 20 μm.

The opposing distinct footpad phenotypes occurring in TOC and *irhom2*^−/−^ imply that the interaction between iRHOM2 and K16 plays a role in the epidermal response to physical stress. To investigate this, wild-type (WT) or TOC iRHOM2-GFP were overexpressed in HaCaT keratinocytes. Fluorescent microscopy showed that the K16 filament network was relatively unchanged in WT-iRHOM2-GFP cells; however, in cells overexpressing TOC mutant-iRHOM2 GFP, K16 showed subtle perinuclear redistribution (Fig. 4a). To recapitulate mechanical footpad stress *in vitro*, CTRL and TOC keratinocytes were cultured in monolayer on flexible membranes and subjected to 4 h of oscillating mechanical stress using the Flexcell system^30^. Both CTRL and TOC cells showed induction of K16 and perinuclear distribution of the filament network. This was more pronounced in TOC cells, which demonstrated significantly increased perinuclear localization of K16 before stress (Fig. 4b,c). *In situ* iRHOM2-K16 PLA of stressed cells demonstrated an intense perinuclear signal, implicating this interaction in K16 filament reorganization (Supplementary Fig. 3b).

K6 anchors K16 to desmosomal adhesion junctions at the cell surface membrane by direct interaction with desmoplakin, resulting in a branch-like filament network, which provides structural support^31^,^32^. Overexpression of either WT or Mut-iRHOM2-GFP in HaCaT keratinocytes led to profound downregulation of K6 (Supplementary Fig. 4a). Moreover, TOC cells subjected to mechanical stress are unable to restore K6 expression, despite upregulation of K16 (Supplementary Fig. 4b). It is therefore tempting to speculate that differential downregulation of K6 in TOC by iRHOM2 releases K16 from desmosomal adhesion structures and facilitates perinuclear reorganization of K16.

Moreover, keratin filament cycling plays a role epithelial migration and proliferation^33^,^34^. In mice, the absence of K6 results in almost doubled K16 filament turnover^29^. To see whether this phenomenon occurred in TOC, we treated keratinocytes with the protein phosphatase inhibitor okadaic acid (OA) for 1 h, which disassembled keratin filaments into cytoplasmic aggregates^35^. Four hours following treatment, CTRL cells show limiting recovery, with few filaments reformed. However, in TOC cells there is partial recovery. Twenty-four hours following treatment with OA, TOC cells show normalization of K16 filaments; however, in CTRL filaments remain aggregated in the cytoplasm (Fig. 4c). K6 filament turnover was also assessed in CTRL cells and did not appear to show accelerated turnover. The low expression of K6 in TOC cells prevented accurate assessment of turnover in these cells (Supplementary Fig 4c). These data reveal that the differential regulation of K16 and K6 in TOC keratinocytes contributes to accelerated filament turnover and perinuclear re-organization of the K16 filament network.

### iRHOM2 regulates migration proliferation and inflammation

**Figure 5:**
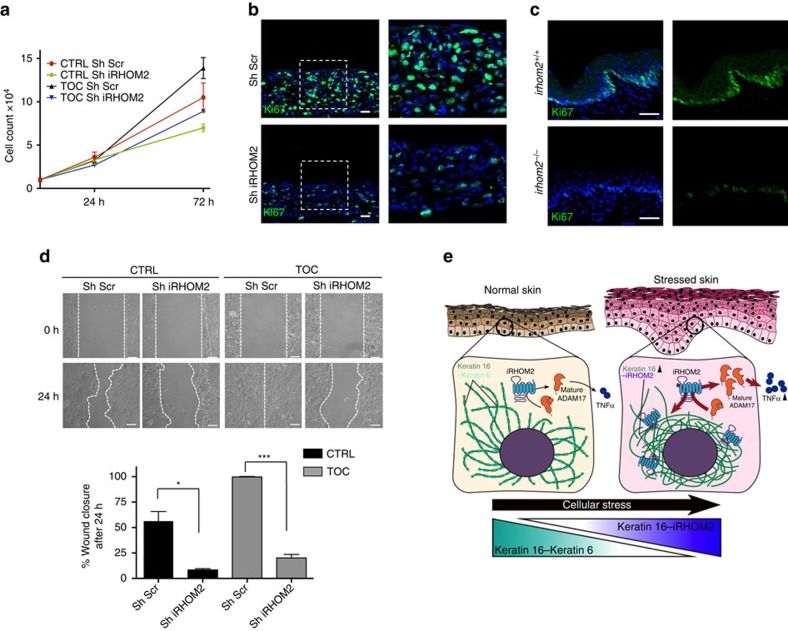
iRHOM2 regulates migration and proliferation. (**a**) Growth curves of cultured cells for Sh-Scr, Sh-IRHOM2 in CTRL and TOC keratinocytes showing slower growth rates for Sh-iRHOM2 cells in both cell lines (mean of three independent experiments). (**b**) Confocal analysis showing the reduction of Ki67 expression in Sh-IRHOM2 TOC 3D human skin equivalents. Scale bars, 20 μm. (**c**) Confocal analysis confirming the reduction of Ki67 expression in the forepaw epidermis of *irhom2*^−/−^ mice. Scale bars, 20 μm. (**d**) Light-microscopy images of CTRL and TOC keratinocyte monolayers 0 and 24 h after scratch-wounding. Graph shows quantification of wound closure, mean of three experiments. Error bars denote s.d. *P<0.05 and ***P<0.001 (Student's *t*-test). Scale bar, 20 μm. (**e**) Model of iRHOM2-mediated regulation of K16 in stressed epithelia.

TOC keratinocytes exhibit increased migration and proliferation rates, similar to that observed in wound edge keratinocytes and epithelial cancers^4^. As K16 is highly expressed at the wound edge^2^,^3^ and also in several epithelial cancers^9^,^14^,^15^, we hypothesized that iRHOM2-K16 could play a role in these cellular events. iRHOM2 was depleted from CTRL and TOC cells using shRNA. The robust downregulation of iRHOM2 and K16 in both cell lines was associated with reduced cell proliferation (Fig. 5a). Ki67, a proliferation marker, was diminished in 3D organotypics derived from shiRHOM2 TOC cells (Fig. 5b) and also in the paws of *irhom2*^−/−^ mice (Fig. 5c). Reduced proliferation and diminished K16 expression could explain the thinner footpads seen in knockout mice.

Scratch-wound assays can demonstrate migration in monolayer^36^. We performed scratch-wound assays using CTRL and TOC cells depleted of iRHOM2 and found that shiRHOM2 resulted in significantly impaired wound closure as a result of abrogated migration (Fig. 5d). Several substrates of ADAM17, activated by iRHOM2, can contribute to migration and proliferation^23^,^24^. It is possible that reduced ADAM17 shedding could in part contribute to the changes in cell behaviour induced by shiRHOM2. We demonstrated that depletion of iRHOM2 in CTRL and TOC keratinocytes results in decreased ADAM17 maturation (Supplementary Fig. 5a) and, consequently, PMA stimulation leads to dampened TNFα release from these cells (Supplementary Fig. 5b). iRHOM2-ADAM17 regulation could explain an unusual feature of the knockout mice phenotype. Despite thinning of the palmoplantar epidermis and barely detectable K16, these mice do not display the PPK-like features observed in K16-null mice, which have a characteristic inflammatory molecular signature^19^,^37^. It might be that reduced ADAM17-mediated shedding of inflammatory cytokines protects *irhom2*^−/−^ mice from developing an inflammatory keratoderma.

## Discussion

K16 is a cytoskeletal protein with unique attributes contributing to its expression in specific pathological and physiological situations. It combines the distinct properties of mechanoresilience, flexibility and dynamic turnover, which are necessary in the hyperproliferative ‘stressed' stratified epithelia where it is expressed^2^,^3^, including at the wound edge, psoriasis, carcinogenesis and the palmoplantar epidermis^1^,^5^,^13^,^14^. However, how this particular keratin is regulated in response to cellular stress has remained elusive. In the present study, using human and mouse models, we report that iRHOM2 is a novel and unexpected regulator of the cytoskeletal response to physical stress through its interaction with K16. We demonstrate direct interaction between iRHOM2 and K16, and show synchronicity between the expression pattern of these two proteins; shRNA-mediated knockdown of iRHOM2 in human keratinocytes or *irhom2*^−/−^ in mouse paw skin is associated with depleted K16 and, reciprocally, high iRHOM2 activity in TOC is associated with increased K16.

Wound edge keratinocytes are poised to undergo proliferation and migration and thus allow tissue regeneration^38^. K16 is rapidly expressed in response to tissue injury and, ultrastructurally, has been shown to adopt a perinuclear arrangement of filaments, which proceeds the onset of migratory behaviour^2^. Further studies have shown that this delocalization is linked to fragmentation of rigid keratin filaments from the cell periphery, thereby increasing cell plasticity, subsequently facilitating proliferation, migration and invasion^39^,^40^. In TOC we observed increased K16 expression and perinuclear distribution of the filament network even in the absence of stress and this was enhanced further by mechanical stretching. We propose that this process is mediated by increased iRHOM2 activity. Our data show increased binding between iRHOM2 and K16 in TOC and this is also linked to downregulation of K16's major binding partner K6. It is tempting to speculate that this results because iRHOM2 and K6 share the same interacting domain with K16, Coil 1B. Intriguingly, we also observed, to a lesser extent, downregulation of K6 at mRNA level; however, how this occurs remains to be explored. Potentially, a negative feedback loop may exist when K6 binding to K16 is reduced, thereby preventing unstable K6 monomer accumulation in the cytoplasm. When K16 and K6 dimerize, the latter binds the complex to desmosomal adhesions structures, stabilizing the intermediate filament network^31^. Therefore, differential downregulation of K6 could release K16 from desmosomes and allow perinuclear localization. Moreover, K16 filament turnover is expedited in the absence of K6 (ref. 29), further facilitating migration and proliferation. Perinuclear localization of K16 and dynamic filament turnover would in turn contribute to the increased proliferation and migration in TOC. The combination of high K16 expression in response to mechanical stress and increased cell turnover in the presence of upregulated iRHOM2 activity provide a possible explanation for the presence of PPK in TOC. This model could also explain the diminished K16 expression and thinner footpads in *irhom2*^−/−^ mice. Although wounded epithelia rapidly induce both K6 and K16, perinuclear reorganization of K16 occurs despite the presence of K6. This also raises the possibility that the differential regulation of K6 and K16 occurring in TOC may be a phenomenon linked to neoplasia in these cells.

As K16 is highly expressed in disease states such as epithelial cancers and inflammatory dermatoses, there may be a broader significance for the role of iRHOM2 in the pathophysiology of these disorders, which remains to be explored. The absence of inflammation in the paws of *irhom2*^−/−^ mice in contrast to K16-KO mice is noteworthy. K16-KO mice have previously been shown to exhibit painful callous formation and activation of inflammatory pathways and, histologically, an influx of inflammatory cells such as macrophages are evident^19^,^37^. However, *irhom2*^−/−^ mice exhibit thinned footpads without overt inflammation. As ADAM17 is a further target of iRHOM2 in keratinocytes and macrophages^22^,^23^,^25^, we suggest that its downregulation in the paws of *irhom2*^−/−^ mice dampens the development of an inflammatory PPK. This could point to a hitherto unidentified role for ADAM17 in the development of PPK-associated inflammation. Thus, pharmacological modulation of iRHOM2 by targeting two stress-response proteins, K16 and ADAM17, could provide a specific therapeutic strategy in the treatment of PPK's, inflammatory epithelial disease and neoplasia.

## Methods

### Antibodies

Details of antibodies used in the following experiments can be found in the Supplementary Information (Supplementary Table 1).

### Statistical analysis

Statistical analyses were carried out using the two-tailed paired Student's *t*-test, asterisks refer to significance in graphs. *P≤0.05, **P≤0.01 and ***P≤0.001.

### Yeast two-hybrid analysis

Yeast two-hybrid screening was performed by Hybrigenics Services, S.A.S., Paris, France (http://www.hybrigenics-services.com).

The coding sequence for human iRHOM2 N terminus (aa 1–403; GenBank accession number gi:306035188) was PCR amplified and cloned into pB29 as an N-terminal fusion to LexA (N-iRHOM2-LexA-C). The construct was validated by sequencing the insert and used as a bait to screen a random-primed human Reconstituted Skin cDNA Library constructed into pP6. pB29 and pP6 derive from the original pBTM116 (refs 41, 42) and pGADGH^43^ plasmids, respectively. One hundred and twelve million clones (13-fold the complexity of the library) were screened using the mating approach with YHGX13 (Y187 ade2-101::loxP-kanMX- loxP-mat) and L40ΔGal4 (mata) yeast strains as previously reported^44^. Twenty-eight His+ colonies were selected by using a medium lacking tryptophan, leucine and histidine. The prey fragments of the positive clones were amplified using PCR and sequenced at both their 5′- and 3′-junctions. The resulting sequences were used to identify the corresponding interacting protein fragments in the GenBank database (NCBI) using an automated procedure. A confidence score was attributed to the interaction, taking into consideration the redundancy and independency of the prey fragments and reading frames, and stop codons in overlapping fragments. Second, taking into account the interactions found in all the previously performed screens at Hybrigenics, using the same library. The scores have been shown to correlate positively with the biological significance of the interaction^45^,^46^

### Cell culture

CTRL and TOC keratinocyte immortalized cell lines previously described^4^ and produced from our laboratory were cultured in DMEM medium (Sigma), which was supplemented with 1% penicillin/streptomycin, 10% FCS, 1% L-glutamine, 1% RM+ supplement (containing EGF). Cells were cultured in a humidified incubator at 10% CO_2_, 37 °C. HaCaT cells were cultured in DMEM supplemented with 10% FCS, 1% L-glutamine and 1% penicillin/streptomycin, and cultured in a humidified incubator at 10% CO_2_, 37 °C.

All experiments were carried out on cells that had been passaged between 10 and 40 times post immortalization.

### Western blotting

Cells were washed twice in PBS and then lysed using lysis buffer (1 M Tris, 2.5 M NaCl, 10% Glycerol, 0.5M Glycerophosphate, 1% Tween-20, 0.5% Nonidet P40, 1 × EDTA-free Complete Protease Inhibitor tablet (Roche) for 15 min on ice. Protein extracts were separated on SDS 12% polyacrylamide gels and transferred to a nitrocellulose transfer membrane. The blots were incubated with specific primary and secondary antibodies and developed according to the manufacturer's instructions (ECL Immobilon, Millipore). Uncut blots are supplied in Supplementary Fig. 6.

### Immunostaining and image processing

Cells plated on cover slips or 5 μm tissue sections were fixed with either 4% paraformaldehyde (PFA) in PBS for 10 min at room temperature, or in ice cold Methanol-Acetone (50:50 mixture) at −20 °C for 10 min depending on the optimal method for detection of the primary antibodies. The following steps were all carried out at room temperature. Cells were permeabilized with 0.1% Triton X-100 for 10 min if PFA fixation was used, then blocked with 5% goat serum for 1 h. Cells or tissue sections were incubated with primary antibody diluted in 5% goat serum overnight at 4 °C. The following day, fixed cells or tissue sections were washed in 3 × 5 min in PBS, then incubated with the appropriate Alexa Fluor (Life Technologies) fluorescent secondary antibody for 1 h at room temperature. The secondary antibody was diluted 1:800 in 5% goat serum. Cells or tissue sections were washed twice 3 × 5 min in PBS, then incubated in 4,6-diamidino-2-phenylindole nuclear stain (1:10,000), then washed twice for 10 min each and finally mounted onto slides and imaged using the Zeiss 710 confocal microscope (Carl Zeiss).

The quantification of perinuclear localization of CK16 filaments was based on the method previously described^47^. CTRL and TOC cells were quantified in ImageJ by drawing a line from the nuclear envelope across the plasma membrane using the freehand tool and plotting the K16-associated fluorescence signal along this line. K16 filament arrangements were classified as ‘perinuclear' when all of the positive filament signals (>50% relative signal intensity, normalized to the maximum fluorescence of the specific cell) were found within 3 μm from the nuclear membrane. Images were selected randomly and blindly subjected to this quantification. For the final bar graph depicting the percentage of perinuclear cells, 90 images for each of CTRL and TOC from three independent experiments each were quantified. Statistical analysis was performed using the unpaired Student's *t*-test.

### Generation of 3D human skin equivalents

Following informed written consent, human dermal fibroblasts were isolated from skin biopsy of a patient undergoing plastic surgery (abdominoplasty, sex and health status are unknown). This procedure was performed under ethical approval of the East London Research Ethics Committee. Fibroblasts were then embedded in 4 mg ml^−1^ collagen 1 matrices at a concentration of 250,000 cells per ml, to produce dermal equivalents. After culturing these dermal equivalents for 24 h, keratinocytes were seeded on top (30,000 cells per cm^2^) and kept in submerged culture for a further 24 h. Afterwards, the human skin equivalents were lifted to the air–liquid interface and cultured until day 14 with alternate daily media change, before being PFA-fixed and paraffin-embedded for processing.

### Transfection of cell lines

RHBDF2 constructs were prepared by subcloning WT RHBDF2 cDNA from Origene clone SC122961 (Origene) into vector pEGFP-N3. Mutant RHBDF2 constructs were prepared using the QuikChange II site-directed mutagenesis kit (Agilent). Details of the primer sequences used to introduce the mutation are available in the Supplementary Information (Supplementary Fig. 8).

N terminus RHBDF2 construct was prepared by cloning WT RHBDF2- N-terminal domain into vector pEGFP-N3. The plasmid was prepared using In-Fusion HD Cloning kit (Clontech Laboratories, Takara). N terminus RHBDF2 constructs were a generous gift from Professor M. Freeman

HaCaT cells were grown on coverslips and plated at a density of 2 × 10^5^ cells per well of a six-well plate. Transfection was carried out after 24 h using. Fugene 6 (Roche). The cell culture medium was changed the next day. Cells were fixed and immunostained 48 h following transfection.

### Co-immunoprecipitation

Cells were grown to confluence in 10 cm plates. Plates were washed twice on ice in ice-cold PBS and then lysis buffer was added with a protease inhibitor cocktail (Roche). Lysates collected after 10 min and pre-cleared by centrifugation at 13,000 G for 10 min at 4 °C. The supernatant was removed from the debris pellet and transferred to a clean tube. Protein concentration was calculated using the Bradford Protein Assay system (Bio-Rad). Fifty microlitres of Dynabeads (Thermo) were prepared according to manufacturer's instructions. Antibodies (2–4 μg) were bound to the beads by incubation at room temperature with rotation for 30 min. Species matched IgG (Rabbit IgG sc-2027, Mouse IgG sc-2025, Santa Cruz) were bound to beads as a control. Two milligram of protein lysate was added to beads and incubated overnight at 4 °C with rotation. The following day, beads were washed three times for 5 min on ice in 700 μl NP-40 wash buffer (50 mM Tris pH 8.0, 150 mM NaCl, 1 mM EDTA and 1% NP40) containing protease and phosphatase inhibitors (Roche) followed by separation on a magnet. Beads were separated from antibody–protein complexes by boiling for 5 min in 2 × Laemelli buffer and the eluate separated by a magnet loaded onto a gel for SDS–PAGE electrophoresis. The lysate was run alongside as an input for the immunoprecipitation.

### Flexcell mechanical stress assay

The Flexcell FX-4000 Tension System (Flexcell International Corporation) is a computer-regulated bioreactor, which uses cyclical vacuum pressure to apply strain to cells cultured on flexible-bottomed culture plates. This was used to subject specific cell monolayers to mechanical stress. Control or TOC cells were grown to near confluence on BioFlex six-well plates coated with pronectin (Flexcell International Corporation), which contain a flexible membrane in each 35 mm well. Each plate was placed over the Flexcell loading station which contains six planar faced posts. Cells were subject to cyclic mechanical stretch with a frequency of 5 Hz and an elongation of amplitude ranging from 10 to 13% based on prior optimization in keratinocytes. Cells were stretched for either 0 h (unstretched control) and 4 h, and then prepared for immunocytochemistry as described above.

### Proximity ligation assay

PLA was performed using the Duolink *in situ* kit (Sigma) according to the manufacturer's instructions, cells were plated on coverslips or Bioflex six-well plates for cell stretching assay (above) and fixed with Methanol Acetone or PFA as described previously. Following fixation, cells were incubated in Duolink blocking solution for 30 min at 37 °C. Previously optimized primary antibodies were diluted in Duolink Antibody diluent and added to cells, which were incubated overnight at 4 °C. The following day the cells were washed in Duolink Wash buffer A 2 × 5 min. The PLA plus and minus probes were diluted 1:5 in antibody diluent and added to the cells, which was incubated for 1hr at 37C. The cells were washed in Duolink Wash buffer A 2 × 5 min. The Ligation–Ligase was prepared according to instructions and applied to cells for 30 min at 37 °C. The cells were washed 2 × 2 min in Duolink wash buffer A and the Duolink Amplification-Polymerase solution added and incubated for 100 min at 37 °C. The cells were washed in Duolink wash buffer B for 2 × 10 min and then mounted with Duolink *in situ* mounting medium with 4,6-diamidino-2-phenylindole before visualization with a Zeiss Confocal 710 microscope (Carl Zeiss). As a negative control, no primary antibodies were applied. A further negative control was the use of iRHOM1 instead of iRHOM2 primary antibody as a positive control, known binding partners (such as K6 and K16) primary antibodies were applied. Quantification was performed using ImageJ software analysis programme and analysed by Student's paired *t*-test using GraphPad Prism (Graphpad, San Diego, USA).

### shRNA lentiviral particle transduction

shRNA lentiviral particle transduction was used to silence RHBDF2 gene expression in human keratinocyte cell lines.

Lentiviral particles were purchased from Santa Cruz biotechnology (Santa Cruz). The product contains a pool of concentrated transduction-ready viral particles containing three target-specific constructs, which encode 19–25 nucleotide (plus hairpin) shRNA designed to knock down gene expression.

Cells were plated at 2 × 10^5^ per well of a six-well plate, which resulted in 50–60% confluence after 24 h. Cells were then treated with Polybrene (Santa Cruz), a cation polymer used to increase the efficiency of infection of cells with the retrovirus. Ten microlitres of Polybrene was added to the cells and incubated at 37 °C for 20 min. The Polybrene media was then removed and to this the lentiviral particles (either RHBDF2 or negative scrambled shRNA sequence control) were added. This was optimized using different concentrations of particles. The cells were incubated overnight at 37 °C in complete media. To select stably transfected cells, Puromycin selection media was used. Cell lysates were obtained as described above and analysed using SDS–PAGE electrophoresis and western blotting for iRHOM2, to determine gene silencing.

### Proliferation and cell migration assays

To measure proliferation, cells were plated at a seeding density of 1 × 10^4^ per well and harvested at 24 and 72 h, and cell counts were performed using the Nucleocounter/Nucleocasette system (ChemoMetec, Denmark). To measure migration, cells were seeded at equal density on a six-well plate and grown for 24–48 h in complete media (as described above) until a confluent monolayer was achieved. Cells were treated with Mitomycin C (400 ng ml^−1^) to prevent proliferation. A vertical scratch was performed using a 200 μl Gilson pipette tip. Cultures were washed three times in PBS and incubated in media. Photographs were taken at 0, 18 and 24 h. Wounded areas were measured using Image J software analysis. Results were analysed by Student's paired *t*-test using Graphpad Prism (Graphpad).

### PMA treatment and ELISA

Cells were plated at equal density (3 × 10^5^ per well) on six-well plates and grown for 24 h in complete media. Cells were subsequently treated with 250 ng ml^−1^ PMA. Media was collected at time points (4 and 24 h) for ELISA. Collected medium specimens were analysed for the TNFα using the DuoSet ELISA kit (R&D Systems, Abingdon, UK) and compared with untreated controls. In all cases, data are presented as mean ±s.e.m. Data shown represents the mean of at least three separate experiments for each cell lines. Results were analysed by Student's paired *t*-test using GraphPad Prism (Graphpad).

### OA treatment

CTRL and TOC keratinocytes were seeded on coverslips in 12-well plates. After reaching the desired confluence, cells were incubated in media containing 1 μM OA (Sigma) for 1 h. The media was replaced without OA and cells were fixed for immunostaining as described above at time points following the treatment (0, 4 and 24 h).

### Animal experiments

All experiments involving mice followed the UK Animal Welfare Act guidelines and approval of the UK Home Office. *irhom2*^−/−^ mice were generated as previously described^22^. All experiments were performed using age and sex-matched littermates.

The fore and hind paw footpad epidermis from 20-week-old gender-matched *irhom2*^−/−^ and *irhom2*^*+/+*^ mice (*n*=4 per genotype) were dissected and fixed in OCT on dry ice. Five micrometre sections were cut and mounted on slides. For histopathological analyses, sections were fixed in 4% PFA and stained with haematoxylin/eosin according to standard protocols. Epidermal thickness was measured using Image J software and analysed by students paired t-test (*n*=4) using GraphPad Prism (Graphpad).

Immunohistochemical staining was performed as previously described and visualized using the Zeiss 710 Confocal Microscope (Carl Zeiss).

### Quantitative real-time PCR

RNA was extracted from cultured cells using the Qiagen RNeasy mini kit (Qiagen, Crawley, UK) following the manufacturer's instructions and converted to cDNA using Invitrogen SuperScript II reverse transcriptase (Invitrogen, Grand Island, NY, USA). Quantitative real-time PCR using TaqMan chemistry was performed on a Rotorgene Q thermocycler (Qiagen) using primers against *KRT6A*, *KRT16* and *GAPDH*, designed in-house, with target sequences across cDNA exon–exon boundaries. Details of the primer sequences used are available in the Supplementary Information (Supplementary Table 2). Specimens were assayed simultaneously for *GAPDH* and K16 or K6A using probes labelled with different fluorophores. The expression of all genes was quantified relative to GAPDH using the Δ*C*_t_ method^48^.

### Data availability

The data that support the findings of this study are available from the corresponding authors (A.C. and D.P.K.) upon reasonable request.

## Additional information

**How to cite this article**: Maruthappu, T. *et al*. Rhomboid family member 2 regulates cytoskeletal stress-associated Keratin 16. *Nat. Commun.* **8**, 14174 doi: 10.1038/ncomms14174 (2017).

**Publisher's note**: Springer Nature remains neutral with regard to jurisdictional claims in published maps and institutional affiliations.

## Supplementary Material

Supplementary Information Supplementary figures and supplementary tables.
