# Supplementary material for: Rhomboid family member 2 regulates cytoskeletal stress-associated Keratin 16
Source: Nat Commun. 2017 Jan 27;8:14174. doi: 10.1038/ncomms14174 (PMC5290154; doi:10.1038/ncomms14174)
Supplement: Supplementary Information — Supplementary figures and supplementary tables. [file ncomms14174-s1.pdf]

Supplementary Fig. 1

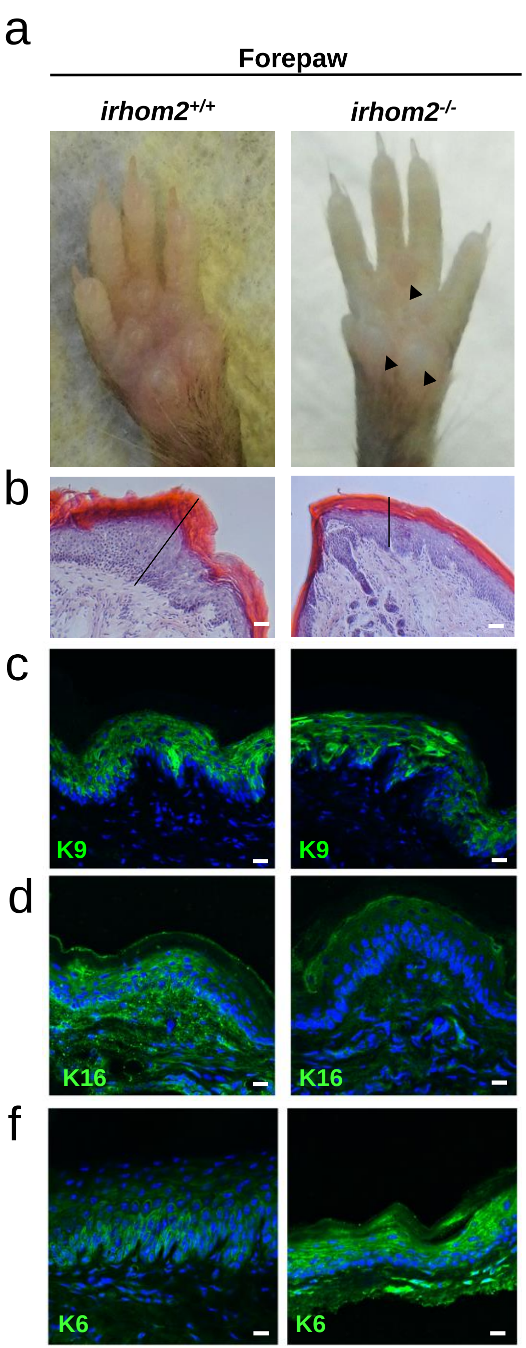

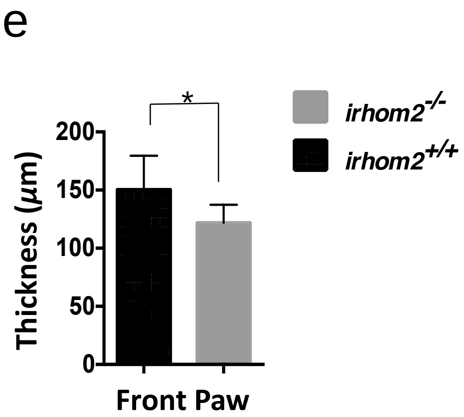

g

Bait	Prey clone library	His+/Ade+/ Laz+ prey clones	True prey clones	K16 fragment prey clones
Human RHBDF2 N-Terminus (aa1-403)	112 x 10^6	28	22	2

Supplementary Figure 1. ***irhom2*** $^{-/-}$ **mice exhibit reduction in epidermal thickness.** (**a**) Photographs of the front paws of 16-week-old *irhom2* $^{-/-}$ and *irhom2* $^{+/+}$ littermates (n=4 per genotype) showing similar features to the hind paws; pallor and diminution of the major stress-bearing paw footpads calluses (black arrowhead) in adult *irhom2* $^{-/-}$ mice. (**b**) Haematoxylin and eosin-stained cross-sections of hind-paw footpads showed a reduction in thickness of the epidermis (black line) in *irhom2* $^{-/-}$ mice. Scale bars: 50 µm. (**c**) Immunohistochemistry showing similar K9 expression in paw skin sections. Scale bars: 20 µm. (**d**) Immunohistochemistry demonstrating reduction of K16 expression in paw skin sections. Scale bars: 20 µm. (**e**) Graph depicts the mean thickness of the epidermis of front paw and back skin calculated from 10 individual measurements in 5 separate cross sections from each genotype (n=3) error bars denote SD, *P<0.05. (**f**) Immunohistochemistry showing upregulation of K6 expression in the front paw and epidermis of *irhom2* $^{-/-}$ mice compared with *irhom2* $^{+/+.}$ (**g**) Table showing the positive prey clones identified by the Yeast 2 Hybrid screen.

Supplementary Fig. 2

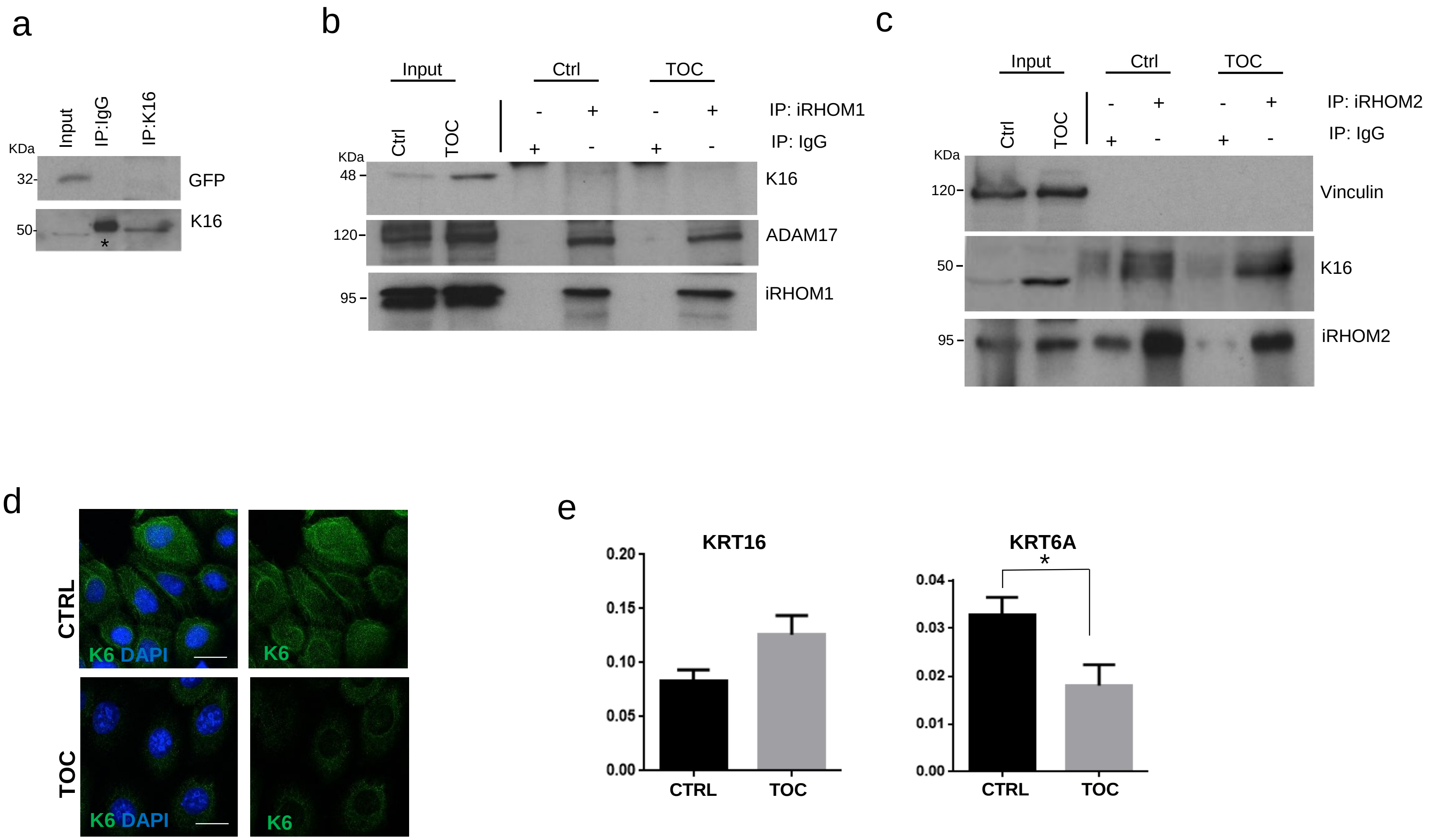

Supplementary Figure 2. iRHOM2 interacts with Keratin 16 and this binding occurs through the N-terminus domain of iRHOM2. (**a**) Cell extracts from CTRL keratinocytes transfected with a vector expressing empty GFP vector were immunoprecipitated with anti-K16 antibody and analyzed by western blot with anti-GFP and anti-K16 antibodies. (**b**) CTRL and TOC keratinocyte lysates were immunoprecipitated with anti-iRHOM1 antibody and analyzed by western blotting with anti-K16, anti-ADAM17 and anti-iRHOM1 antibody. (**c**) CTRL and TOC keratinocyte lysates were immunoprecipitated with anti-iRHOM2 antibody and analyzed by western blotting with anti-K16, anti-iRhom2 and anti-Vinculin antibody. (**d**) Confocal analysis showing reduced K6 expression in TOC keratinocytes. Scale Bar: 20µm. (**e**) Graphs depicting fold change of mRNA expression of K16 and K6 in CTRL and TOC keratinocytes measured by qPCR. (n=3) error bars denote SD, *P<0.05.

a

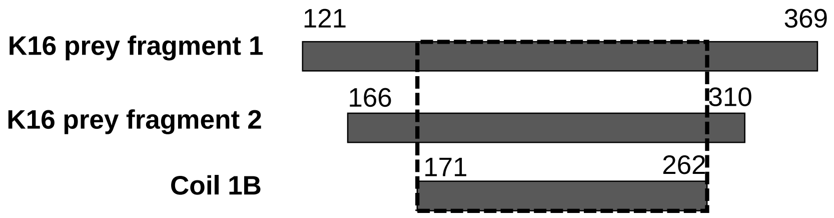

[1]MTTCSRQFTSSSSMKGSCGIGGGIGGGSSRISSVLAGGSCRAPSTYGGGLSVSSR
FSSGGACGLGGGYGGGFSSSSSFGSGFGGGYGGGLGAGFGGGLGAGFGGGFAG
GDGLLVGSEKVT**MQNLNDRLASYLDKVRALEEANADLEVKIRDWYQRQRPSEIKD**
YSPYFKTIEDLRNKIIAATIENAQPILQIDNARLAADDFRTKYEHELALRQTVEADVN
GLRRVLDELTLARTDLEMQIEGLKEELAYLRKNHEEEMLALRGQTGGDVNVEMDA
APGVDLSRILNEMRDQYEQMAEKNRRDAETWFLSKTEELNKEVASNSELVQSSRS
EVTELRRVLQGLEIELQSQLSMKASLENSLEETKGRYCMQLSQIQGLIGSVEEQLA
QLRCEMEQQSQEYQILLDVKTRLEQEIATYRRLLEGEDAHLSSQQASGQSYSSREV
FTSSSSSSSRQTRPILKEQSSSSFSQGQSS[473]

b

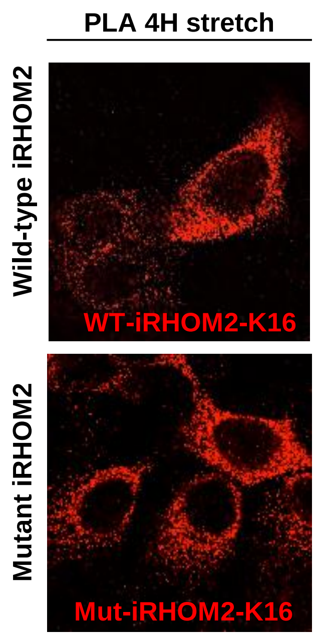

Supplementary Figure 3. iRHOM2 regulates the reorganization and dynamicity of K16 filaments. (**a**) K16 prey fragments identified by the Yeast 2 Hybrid screen, both of which span the Coil 1B domain of K16. Below, K16 amino acid sequence. Bold letters refer to identified prey region starting from amino acids 167-310, underlined letters correspond to the Coil 1B domain. (**b**) PLA of GFP (green) and K16 (red) in cells overexpressing either WT-iRHOM2-GFP or TOC-mutant-iRHOM2-GFP following 4H cyclical stretching. Images analysed by confocal microscopy. Scale Bar 20µm.

Supplementary Fig. 4

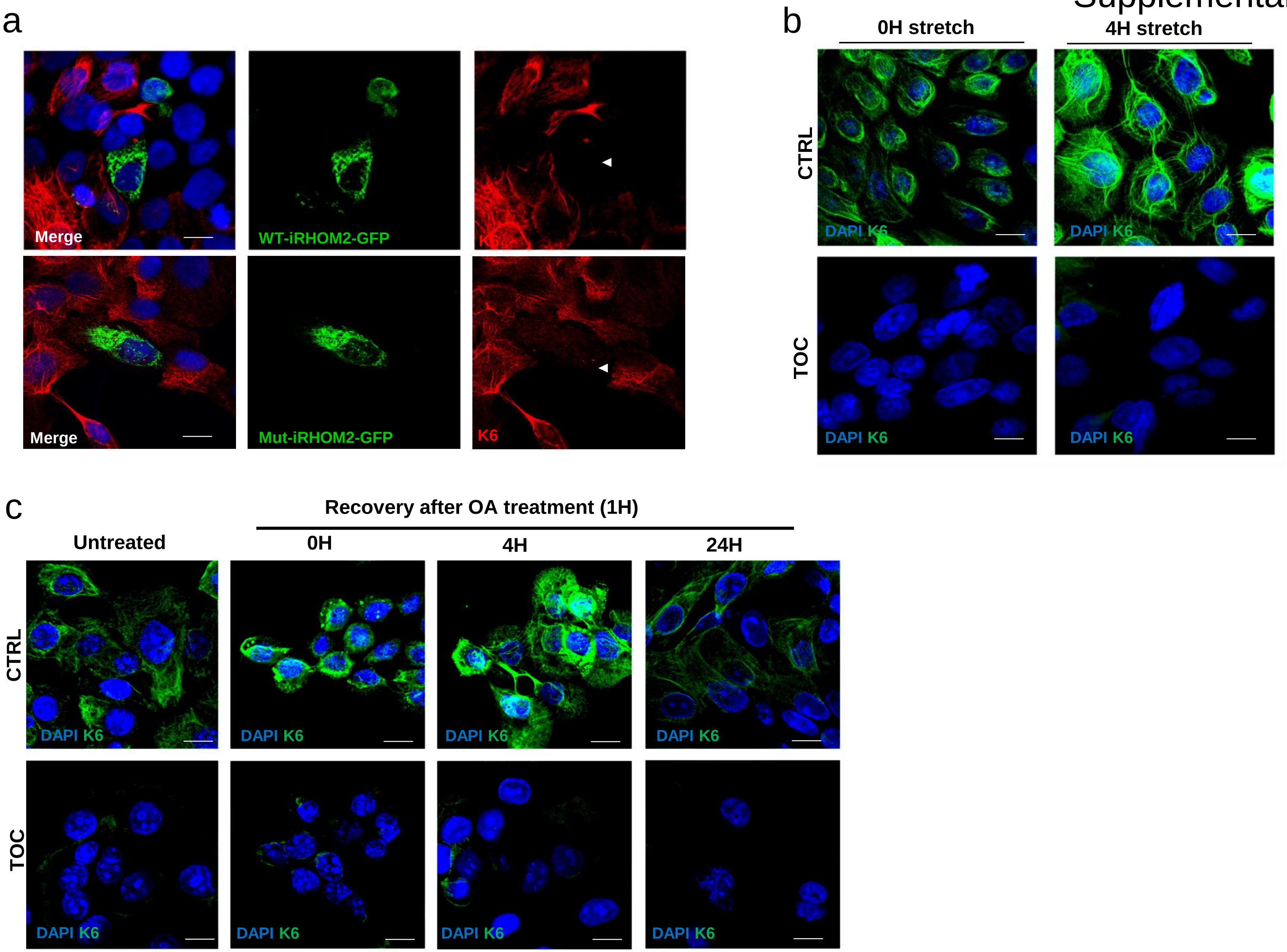

Supplementary Figure 4. iRHOM2 differentially regulates K16 and K6. (**a**) CTRL keratinocytes were transfected with either WT-iRHOM2-GFP or TOC Mutant-iRHOM2-GFP and immunostained for endogenous K6 and analyzed by confocal microscopy. K6 expression is reduced in transfected cells (white arrowhead) Scale Bar: 20µm. (**b**) CTRL and TOC keratinocytes were subjected to cyclical mechanical stretch at a frequency of 5hz and amplitude 10-13% using Flexcell FX-4000 Tension system for 0h and 4hrs respectively. Stretched cells were immunostained for K6 and and analyzed by confocal microscopy. Scale Bar: 20µm. (**c**) Confocal analysis of CTRL and TOC keratinocytes treated with 1µM Okadaic Acid for 1 hour, then fixed 4H and 24H following treatment and immunostained for K6. White arrowheads represent aggregated K16 filaments. Scale Bar: 20µm.

a

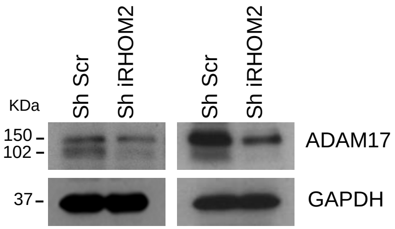

b

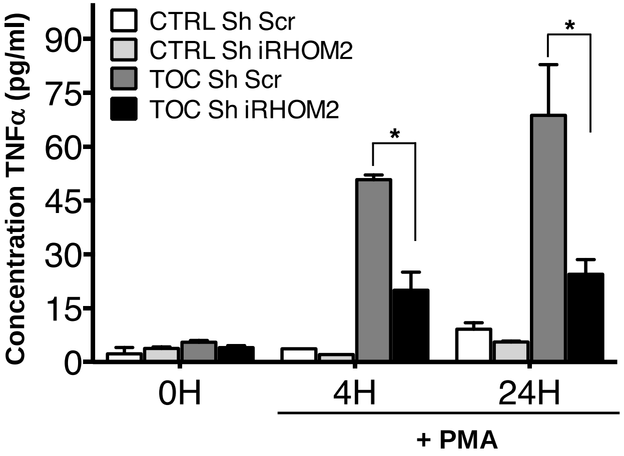

Supplementary Figure 5. iRHOM2 regulates inflammation. (**a**) Western blot analysis showing the modulation of ADAM17 in normal and TOC shiRHOM2 keratinocytes. GAPDH is used as a loading control. (**b**) Reduced shedding of ADAM17 substrate TNFα measured by ELISA in CTRL and TOC keratinocytes depleted of iRHOM2 (shiRHOM2) after stimulation with 250ng/ml PMA for 0, 4 and 24 hours. (n=3) Error bars denote SD **P*<0.05.

Supplementary Fig. 6

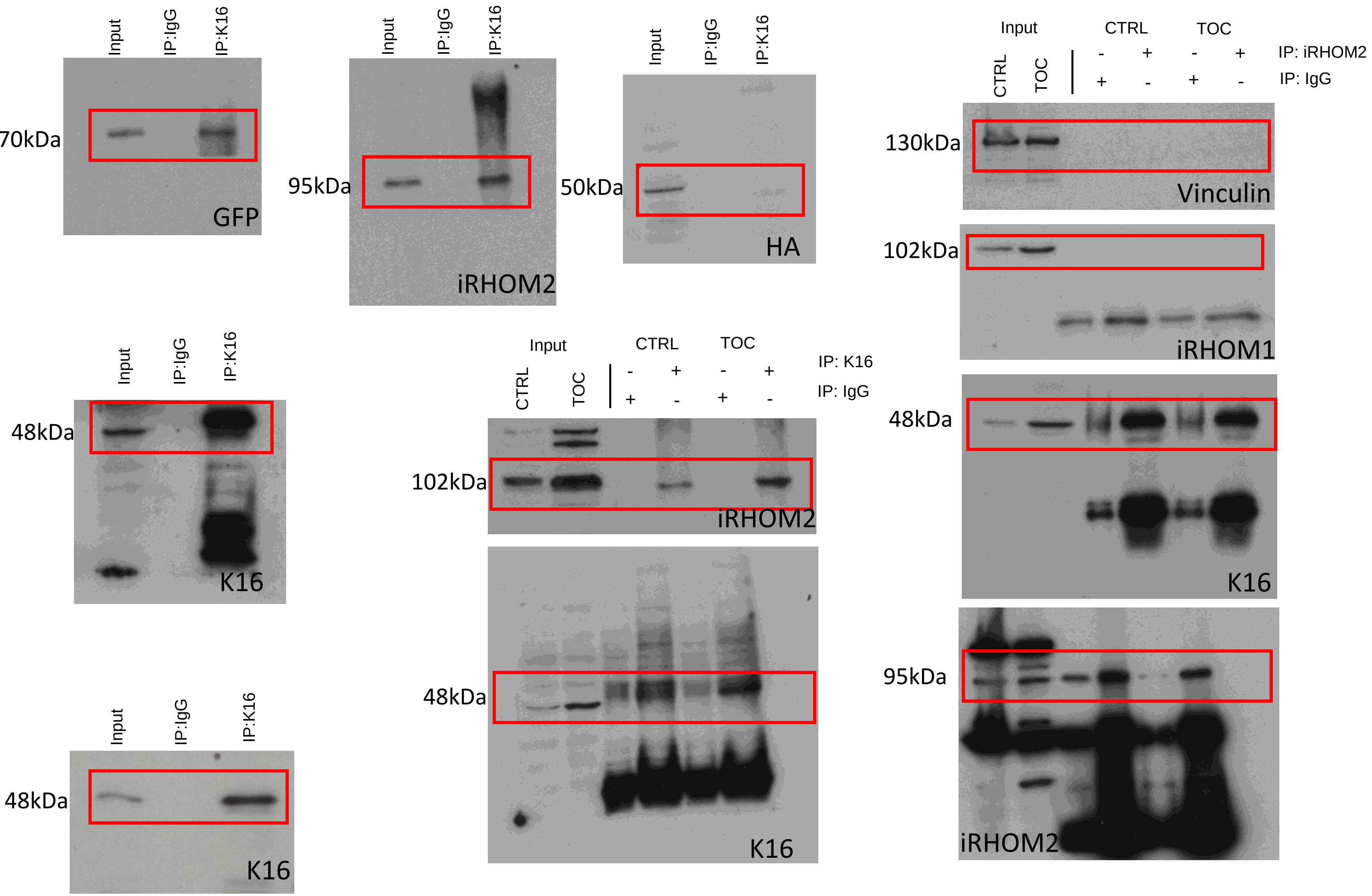

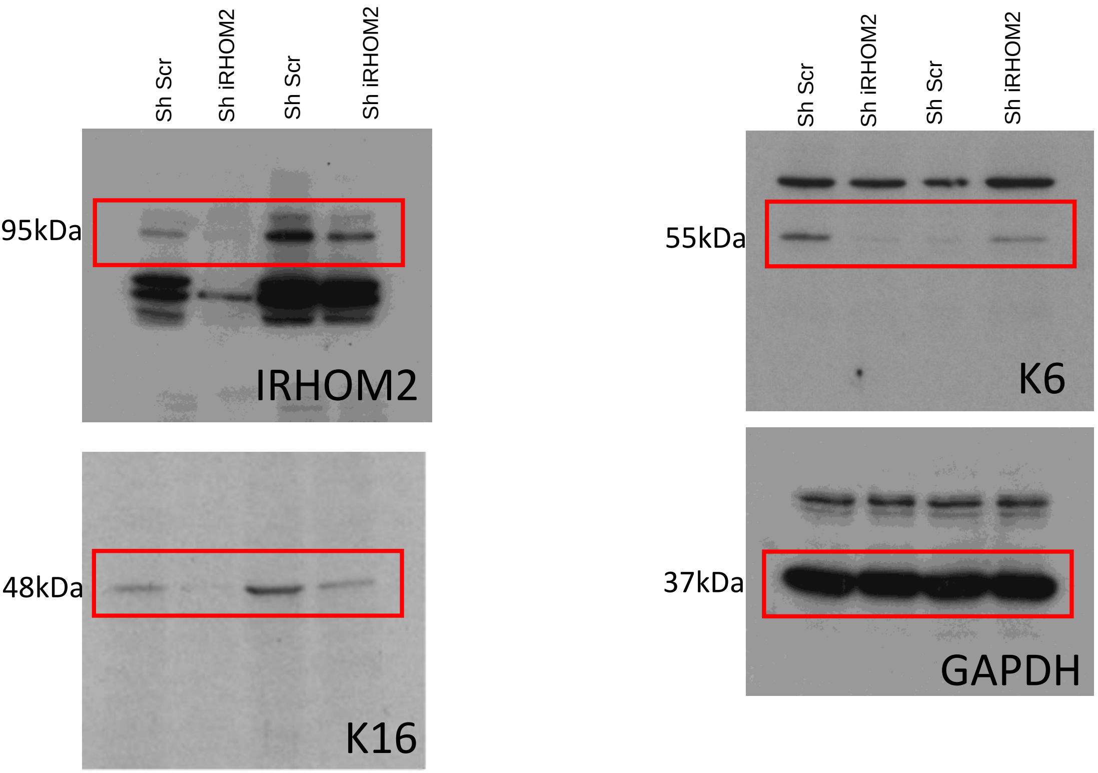

Supplementary Figure 6. Uncropped images of the most important western blots.

Supplementary Table 1

Antibody	Reference	WB Dilution	ICC/IHC Dilution
RHBDF2 (Rabbit)	HPA018080 (Sigma)	1:500	1:50
RHBDF1 (Rabbit)	Ab81342 (Abcam)	X	1:100
Cytokeratin 16 (Mouse)	LL0025 (Cancer Research UK)	1:500	1:50
Cytokeratin 16 (Rabbit)	Ab76416 (Abcam)	1:500	1:50
Cytokeratin 16 (Rabbit)	Ab182791 (Abcam)	1:500	1:100
Cytokeratin 6 (Mouse)	Ab18586 (Abcam)	X	1:100
Cytokeratin 9 (Rabbit)	gift of D Leslie-Pedrioni (University of Dundee, UK)	X	1:250
Ki-67 (Rabbit)	Ab15580 (Abcam)	X	1:100
Anti-HA (Rat)	3F10 (Roche)	1:500	X
ADAM17	Ab2051 (Abcam)	1:500	X
Vinculin	Ab18058 (Abcam)	1:80000	X
GAPDH	Ab8245 (Abcam)	1:2000	X
Anti-GFP (Mouse)	Ab290 (Abcam)	1:500	1:200

Supplementary Table1. Table showing details of antibodies used and their working concentrations.

RHBDF2 TOC Primers for Site Directed Mutagenesis

RHBDF2-SDM-TOC F	5’-GCAAGATGCCCAAGAcTGTGGATCCGCTGGC-3’
RHBDF2-SDM-TOC R	5’- GCCAGCGGATCCACAgTCTTGGGCATCTTGC -3’

Keratin 6 and 16 Primers for qPCR

Keratin 6A F	5’-CCCGAGCCTGATTCCTAGTCC -3’
Keratin 6A R	5’-GAGTTGGCACTGAAACCCCG -3’
Keratin 16 F	5’-CTTCCCGCGAGGTCTTCAC -3’
Keratin 16 R	5’-GGCAGCTCAGTTCTAGGAGC -3’

Supplementary Table 2. Primer sequences for site directed mutagenesis and qRT-PCR of K6 and K16.
